# *Stenotrophomonas maltophilia* bacteremia in children: risk factors and mortality rate

**DOI:** 10.1186/s13756-021-00888-w

**Published:** 2021-01-22

**Authors:** Mohammed Alsuhaibani, Alanoud Aljarbou, Sahar Althawadi, Abdulrahman Alsweed, Sami Al-Hajjar

**Affiliations:** 1 Department of Pediatrics, College of Medicine, Qassim University, Qassim, 51452 Saudi Arabia; 2 Department of Pediatrics, College of Medicine, Imam Mohammad Ibn Saud Islamic University, Riyadh, Saudi Arabia; 3 Department of Pathology and Laboratory Medicine, Microbiology Laboratory, King Faisal Specialist Hospital and Research Center, Riyadh, Saudi Arabia; 4 Department of Pediatrics, King Faisal Specialist Hospital and Research Center, Riyadh, Saudi Arabia

**Keywords:** Trimethoprim-sulfamethoxazole, *Stenotrophomonas maltophilia*, Bacteremia, Bloodstream infections, Children

## Abstract

**Purpose:**

*Stenotrophomonas maltophilia* (*S. maltophilia*) is an opportunistic and nosocomial pathogen that can cause an invasive and fatal infection, particularly in hospitalized and immunocompromised patients. However, little is known about the impact of *S. maltophilia* bacteremia in pediatric patients. Therefore, we aimed to identify risk factors for mortality, antibiotics susceptibility to *S. maltophilia*, and mortality rates in pediatric patients with *S. maltophilia* bacteremia.

**Methods:**

We conducted a retrospective cohort study by identifying all *S. maltophilia* positive blood cultures in the microbiology laboratory database between January 2007 and December 2018 from hospitalized pediatric patients (age 1–14 years). After identifying patients with *S. maltophilia* bacteremia, medical charts were reviewed for demographics, clinical data, and outcomes within seven days of bacteremia diagnosis. Risk factors associated with mortality in *S. maltophilia* bacteremia patients were determined using univariate and multivariate analyses.

**Findings:**

Sixty-eight pediatric patients with *S. maltophilia* bacteremia were identified. All infections were nosocomial infections, and (88.2%) bacteremia cases were catheter-related bloodstream infections. On multivariate analysis, ICU admission prior to bacteremia episode and neutropenia were the major risk factors associated with mortality. *S. maltophilia* was the most susceptible to trimethoprim and sulfamethoxazole (TMP/SMX, 94.1%), followed by levofloxacin (85.7%). The overall mortality rate within seven days of *S. maltophilia* bacteremia diagnosis was 33.8%.

**Conclusion:**

*S. maltophilia* bacteremia is a devastating emerging infection associated with high mortality among hospitalized children. Therefore, early diagnosis and prompt management based on local susceptibility data are crucial. Various risk factors, especially ICU admission prior to bacteremia episode and neutropenia, are associated with *S. maltophilia* bacteremia mortality.

## Introduction

*Stenotrophomonas maltophilia* (*S. maltophilia*) is a non-fermenting, gram-negative bacillus that can cause opportunistic infections, especially in hospitalized and immunocompromised patients [1, 2] This organism could lead to invasive diseases, such as pulmonary, urinary, gastrointestinal, and soft tissue infections. Additionally, meningitis and endocarditis have been reportedly caused by *S. maltophilia* infections [3–6]. *S. maltophilia* has also been recognized as a cause of bacteremia, particularly in intensive care units (ICUs) and among immunocompromised patients. Central venous catheters, using antibiotics, particularly meropenem, lengthy hospitalization, and malignancy have been described as risk factors for *S. maltophilia* bacteremia [7, 8].

The intrinsic resistance of *S. maltophilia* to multiple antibiotics, including cephalosporins and meropenem, which are commonly used for empiric therapy, makes it a therapeutic challenge. Trimethoprim/sulfamethoxazole (TMP/SMX) is the most effective antibiotic for *S. maltophilia* treatment, but resistance has been reported [9, 10].

*S. maltophilia* strains are resistant to several antibiotics, which make it a treatment challenge and life-threatening infection. Mortality can be as high as 69% but varies according to the associated risk factors [11, 12] in Saudi Arabia, Studies describing *S. maltophilia* bacteremia in children are limited [13, 14]. This study aimed to identify the risk factors for mortality from *S. maltophilia* bacteremia, determine the antibiotic susceptibility profile of *S. maltophilia*, and determine the mortality rate in children with *S. maltophilia* bacteremia.

## Methods

This study was a retrospective chart review of children who had been admitted to King Faisal Specialist Hospital and Research Center (KFSHRC) in Riyadh with *S. maltophilia* bacteremia. KFSHRC is a tertiary care center with transplantation and oncology services. This study was approved by the KFSHRC Institutional Review Board.

All *S. maltophilia* positive blood cultures between January 2007 and December 2018 among hospitalized children ≤ 14 years old were included in this study. We identified *S. maltophilia*-positive blood cultures from the microbiology laboratory database then clinical records were reviewed. Bacteremia was defined as ≥ one positive blood culture from a central line or a peripheral blood sample with clinical symptoms [15, 16]. Asymptomatic patients with positive blood cultures whose *S. maltophilia* bacteremia resolved without treatment (confirmed by negative culture) were excluded because of the possibility of contamination. Hospital-acquired *S. maltophilia* infection was determined by an isolate recovered from blood culture 48 h after admission to the hospital [17–19]. Furthermore, isolation of additional bacteria species from the initial blood culture was considered a polymicrobial infection.

We collected the following clinical data: primary diagnosis; admission to the ICU or intubation prior to the bacteremia episode; prior or concomitant use of antibiotics, steroids, chemotherapy, or immunosuppressive therapy; and neutropenia (total granulocyte count < 1000/mL) within the 14 days preceding infection. Additionally, the presence of a central line catheter, clinical manifestations and complete blood counts on the first day of bacteremia were recorded. The mortality was defined as death taking place within 7 days from an *S. maltophilia*-positive blood culture, regardless of comorbidities [7, 20].

All cultures were analyzed using local hospital and laboratory protocols. From 2007 to 2015, the automated BacTec 9240 (Becton Dickinson, Sparks, MD, USA) was used for blood counts. From 2016 to 2018, BACT/ALERT® VIRTUO^®^ blood culture detection system (bioMérieux, Marcy l’Étoile, France) was used. *S. maltophilia* was identified and susceptibility testing was performed using a VITEK^®^ 2 (bioMérieux). Antibiotic susceptibility testing was performed using Gram-Negative Card 292. Interpretation of zone diameters for classification as susceptible, intermediate, or resistant was based on the Clinical and Laboratory Standards Institute guidelines (CLSI M100) [21].

Both descriptive and inferential statistics were used for data analysis. Statistical analysis was carried out using Statistical Package for the Social Sciences (SPSS) version 21.0 (IBM Corp, Armonk, NY, USA). The variables were taken as significant at a *p* value ≤ 0.05. The associations between independent variables (baseline characteristics, i.e., sex, age, primary diagnosis, malignancy, ICU admission prior to episode, and types of antibiotics) and dependent variables (the outcome), were tested using Chi-square tests. Multivariate regression analysis was used to determine factors independently associated with the outcome of the mortality, and the results were reported as adjusted odds ratios with 95% CI.

## Results

Seventy-two pediatric patients with *S. maltophilia* bacteremia were identified. Of the 72 cases, 4 patients were excluded: 1 patient was older than 14 years, and 3 patients had bacteremia that resolved without treatment. The patient demographic and clinical characteristics, and the outcomes are shown in Table 1. Of the remaining 68 patients, 34 (50%) were males. The median age was 21.5 months (IQR 5.5–81). The patients' ages were classified as follows: ≤ 12 months 26 (38.2%); 13–36 months 21 (30.9%); and > 36 months 21 (30.9%), and all the patients developed the bacteremia as hospital-acquired infections.

**Table 1:** Demographic and etiologic characteristics of patients with *S. maltophilia* bacteremia

Parameters	Overall n (%) (n = 68)	Death n (%) (n = 23)	Resolved n (%) (n = 45)	*p* value^§^
Sex				
Male	34 (50.0%)	13 (56.5%)	21 (46.7%)	0.442
Female	34 (50.0%)	10 (43.5%)	24 (53.3%)	
Age in months				
≤ 12	26 (38.2%)	7 (30.4%)	19 (42.2%)	0.639
13–36	21 (30.9%)	8 (34.8%)	13 (28.9%)	
> 36	21 (30.9%)	8 (34.8%)	13 (28.9%)	
Primary diagnosis				
Malignancy	20 (29.4%)	7 (30.4%)	13 (28.9%)	0.895
Anemia	10 (14.7%)	2 (8.7%)	8 (17.8%)	0.317
Cardiac	11 (16.2%)	5 (21.7%)	6 (13.3%)	0.373
Primary immunodeficiency	8 (11.8%)	2 (8.7%)	6 (13.3%)	0.574
Metabolic	5 (7.4%)	2 (8.7%)	3 (6.7%)	0.762
HLH	6 (8.8%)	3 (13.0%)	3 (6.7%)	0.380
Renal	3 (4.4%)	1 (4.3%)	2 (4.4%)	0.985
Other	5 (7.4%)	1 (4.3%)	4 (8.9%)	0.497

^§^*p* value has been calculated using Chi-square test

^**^Significant at *p* ≤ 0.05

The most common underlying primary diagnosis was malignancy 20 (29.4%), congenital heart diseases 10 (16.2%), anemia (11, 14.7%), and primary immunodeficiency 8 (11.8%). There were 22 patients (32.4%) who had had a transplant, 20 patients (29.4%) were on chemotherapy, and 19 patients (27.9%) were on steroids. The majority of the patients had a central line catheter (97%), and most of the bacteremia was catheter-related bloodstream infection (88.2%).

**Fig. 1:**
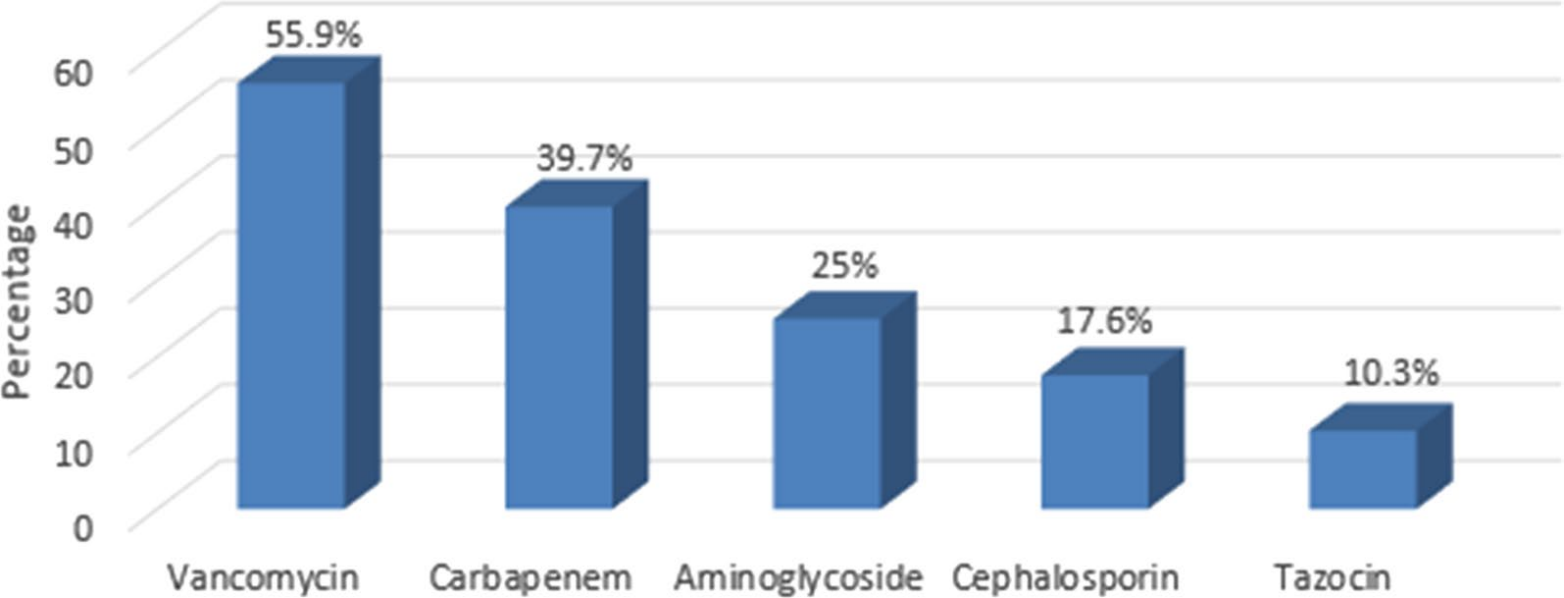
Antibiotics used prior to *S. maltophilia* episodes

The most common clinical presentation of *S. maltophilia* bacteremia, fever, was present in 67.6% of patients, followed by respiratory symptoms (38.2%). Positive respiratory culture with *S. maltophilia* was detected in 24 patients (35.3%), and polymicrobial blood culture was found in 21 patients (30.9%). Polymicrobial blood culture included 60% gram-negative (*Enterobacter, Acinetobacter, Pseudomonas, and Klebsiella* species), 20% gram-positive (*Enterococcus, Coagulase-negative Staphylococcus, and Streptococcus* species), and 20% candida species. The most common antibiotics taken within 14 days prior to the *S. maltophilia* bacteremia diagnosis were vancomycin then meropenem Fig. 1.

**Fig. 2:**
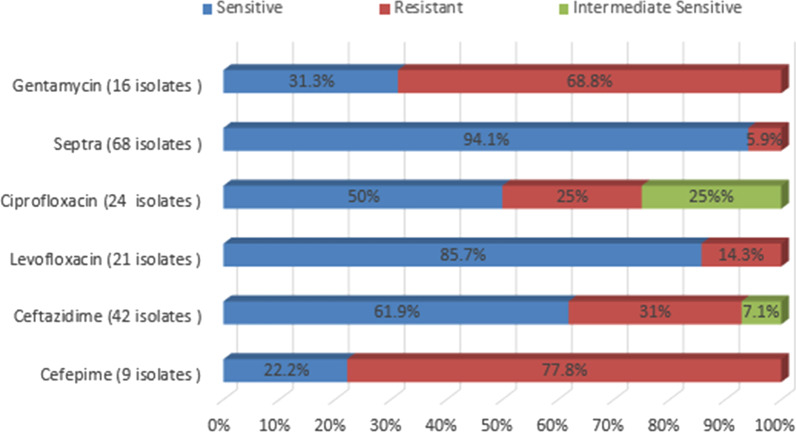
Antibiotic susceptibility of *S. maltophilia* blood isolates

Antibiotic susceptibility to TMP/SMX was the highest (94.1%), followed by levofloxacin (85.7%), while ceftazidime and ciprofloxacin susceptibilities were 61.9% and 50% respectively, as shown in Fig. 2. The antibiotic susceptibility of the *S. maltophilia* blood isolates is shown in Fig. 2. The risk factors associated with mortality as determined by univariate analysis are shown in Table 2. Using multivariate regression analysis, we found that ICU admission prior to episode and neutropenia were the risk factors independently associated with mortality, as shown in Table 3. There were no statistically significant differences in mortality in patients received TMP/SMX as monotherapy (36 patients) and (11 patients) who received it in combination with other antibiotics (fluoroquinolone, ceftazidime, or an aminoglycoside). The overall mortality rate within 7 days of *S. maltophilia* bacteremia diagnosis was 33.8%.

**Table 2:** Univariate analysis for factors associated with mortality of patients with *S. maltophilia* bacteremia

Factor	Overall n (%) (n = 68)	Death n (%) (n = 23)	Resolved n (%) (n = 45)	*p* value^§^
ICU admission prior to episode				
Yes	28 (41.2%)	19 (82.6%)	9 (20%)	< 0.001**
No	40 (58.8%)	4 (17.4%)	36 (80%)	
Intubation prior to episode				
Yes	23 (33.8%)	14 (60.9%)	9 (20.0%)	
No	45 (66.2%)	9 (39.1%)	36 (80.0%)	
Immunosuppressive therapy				
Yes	22 (32.4%)	7 (30.4%)	15 (33.3%)	0.809
No	46 (67.6%)	16 (69.6%)	30 (66.7%)	
Chemotherapy				
Yes	20 (29.4%)	7 (30.4%)	13 (28.9%)	0.895
No	48 (70.6%)	16 (69.6%)	32 (71.1%)	
Received meropenem				
Yes	27 (39.7%)	15 (65.2%)	12 (26.7%)	
No	41 (60.3%)	8 (34.8%)	33 (73.3%)	
Organ transplant				
Yes	22 (32.4%)	6 (26.1%)	16 (35.6%)	0.430
No	46 (67.6%)	17 (73.9%)	29 (64.4%)	
Steroid therapy				
Yes	19 (27.9%)	7 (30.4%)	12 (26.7%)	0.743
No	49 (72.1%)	16 (69.6%)	33 (73.3%)	
Neutropenia				
Yes	32 (47.1%)	16 (69.6%)	16 (35.6%)	
No	36 (52.9%)	7 (30.4%)	29 (64.4%)	
Central line				
Yes	66 (97.1%)	23 (100.0%)	43 (95.6%)	0.305
No	2 (2.9%)	0	2 (4.4%)	
Platelet count				
< 150	56 (82.4%)	23 (100%)	33 (73.3%)	
≥ 150	12 (17.6%)	0	12 (26.7%)	
Positive respiratory culture				
Yes	24 (35.3%)	17 (73.9%)	7 (15.6%)	< 0.001**
No	44 (64.7%)	6 (26.1%)	38 (84.4%)	
Treatment with TMP/SMX				
Monotherapy	36 (76.6%)	11 (73.3%)	25 (78.1%)	0.718
Combination	11 (23.4%)	4 (26.7%)	7 (21.9%)	

^§^*p* value has been calculated using Chi-square test

**Significant at *p* ≤ 0.05

**Table 3:** Multivariate regression analysis to predict the influence of non-survival rates from the baseline characteristics of participants (n = 68)

Factor	AOR	95% CI	*p* value^§^
ICU admission			
Yes	Ref		
No	0.036	0.003–0.406	
Intubation before episodes			
Yes	Ref		0.802
No	0.742	0.072–7.632	
Prior use of antibiotics			
Yes	Ref		0.844
No	1.275	0.114–14.294	
Received meropenem			
Yes	Ref		0.080
No	0.242	0.049–1.185	
Had neutropenia			
Yes	Ref		
No	0.083	0.013–0.537	

*AOR* adjusted odds ratio, *CI* confidence interval

**Significant at *p* ≤ 0.05 level

## Discussion

*S. maltophilia* bacteremia is a relatively rare but life-threatening infection, causing significant mortality. In our study, we described the risk factors for mortality in 68 pediatric patients with *S. maltophilia* bacteremia.

Though *S. maltophilia* is found in the community [7], in our study, all infections were a hospital-acquired infection. Multiple risk factors for mortality have been identified in previous studies of *S. maltophilia* infections in children, including prior use of antibiotics, neutropenia, mechanical ventilation, ICU stays, and malignancy [22–24]. Also, ICU admission showed to be a risk factor for *S. maltophilia* bacteremia acquisition [25]. In our study, we documented that ICU admission prior to bacteremia episode and neutropenia are risk factors for mortality in patients with *S. maltophilia* bacteremia. Approximately half of patients in this study were neutropenic, which may be explained that our center is specialized in immunocompromised and oncology patients.

In the current study, we found that polymicrobial infection was most often caused by other gram-negative bacteria (30.9%) *Enterobacter, Acinetobacter, Pseudomonas, and Klebsiella* species. Sattler et al. also reported that gram-negative co-infections with *Acinetobacter* and *Enterobacter* species were common among children with *S. maltophilia* bacteremia but found a much higher prevalence of polymicrobial infection (70% vs. 30.9%) [7].

The management of *S. maltophilia* bacteremia and sepsis is challenging because of the bacteria's extensive intrinsic and induced antimicrobial resistance [26]. Different molecular mechanisms of resistance have been documented and include ß-lactamase production against ß-lactam antibiotics, multidrug efflux pumps, the plasmid-encoded qnr gene against quinolones, and the presence of class 1 integrons, known to be responsible for resistance to TMP/SMX [27]. According to SENTRY antimicrobial surveillance data and other literature reviews of bacteremia, most bacterial isolates are highly susceptible to TMP/SMX and levofloxacin, with low to moderate sensitivity to ceftazidime and ciprofloxacin. These data are consistent with the sensitivity pattern among our *S. maltophilia* isolates [28, 29]. Although there is developing resistance to TMP/SMX, the prevalence of resistance in our isolates was very low (5.9%) which made it the best empirical treatment for our center [30]. Furthermore, we did not find a statistically significant difference in mortality in patients who received TMP/SMX as monotherapy and those who received it in combination with ceftazidime, aminoglycoside or fluoroquinolones. Tokatly et al. advocated the use of a combination of TMP/SMX and ciprofloxacin or minocycline in critically ill children [24]. However, the best empirical or combination treatment for *S. maltophilia* needs further study.

Mortality related to bacteremia in children has been reported to be high, with crude mortality rates ranging from 14 to 69% [31, 32]. A 5-year multicenter retrospective study of critically ill children with *S. maltophilia* bacteremia, conducted between 2012 and 2017, found a crude mortality rate of 42%, although the mortality attributed to infection was 18% [24]. Our study revealed a 7-day crude mortality rate of 33.8%, but this decreased to 26.4% if polymicrobial infections were excluded. A similar study conducted in adults found a crude mortality rate of 47.5% among patients with *S. maltophilia* infection and an eightfold increase in mortality risk with an attributable mortality rate of 26.7% due to bacteremia caused by *S. maltophilia* [33].

Our study's limitations are its retrospective design, single-center location, the lack of appropriate control groups, and the clinical status of our patients (with many being immunocompromised or in critical care).

This study revealed that *S. maltophilia* bacteremia is an emerging fatal infection associated with high mortality among children. Therefore, early diagnosis and prompt management based on local data susceptibility are crucial. It identified various risk factors associated with *S. maltophilia* bacteremia mortality, of which ICU admission prior to bacteremia episode and neutropenia were associated with the highest risk. A multicenter, prospective cohort study is needed to confirm these risk factors associated with *S. maltophilia* mortality in children.

## Data Availability

The data and material of the study are available from the corresponding author MA on request.
